# Microelectrochemical Smart Needle for Real Time Minimally Invasive Oximetry

**DOI:** 10.3390/bios10110157

**Published:** 2020-10-29

**Authors:** Daniela Vieira, Francis McEachern, Romina Filippelli, Evan Dimentberg, Edward J Harvey, Geraldine Merle

**Affiliations:** 1 Experimental Surgery, Faculty of Medicine, McGill University, Montreal, QC H3A 0C5, Canada; daniela.vieira@mail.mcgill.ca (D.V.); francis.mceachern@mail.mcgill.ca (F.M.); romina.filippelli@mail.mcgill.ca (R.F.); Evan.dimentberg@mail.mcgill.ca (E.D.); 2 Department of Surgery, Faculty of Medicine, McGill University, Montreal, QC H3A 0C5, Canada; edward.harvey@mcgill.ca; 3 Chemical Engineering Department, Ecole Polytechnique de Montréal, P.O. Box 6079 Station, Montreal, QC H3C 3A7, Canada

**Keywords:** oxygen biosensor, spin dipping, laccase, acupuncture needles, nanomaterials

## Abstract

A variety of brain disorders such as neural injury, brain dysfunction, vascular malformation, and neurodegenerative diseases are associated with abnormal levels of oxygen. Current methods to directly monitor tissue oxygenation in the brain are expensive and invasive, suffering from a lack of accuracy. Electrochemical detection has been used as an invasiveness and cost-effectiveness method, minimizing pain, discomfort, and injury to the patient. In this work, we developed a minimally invasive needle-sensor with a high surface area to monitor O_2_ levels in the brain using acupuncture needles. The approach was to directly etch the iron from stainless steel acupuncture needles via a controlled pitting corrosion process, obtaining a high microporous surface area. In order to increase the conductivity and selectivity, we designed and applied for the first time a low-cost coating process using non-toxic chemicals to deposit high surface area carbon nanoparticle, catalytically active laccase, and biocompatible polypyrrole. The physicochemical properties of the materials were characterized as well as their efficacy and viability as probes for the electrochemical detection of PO_2_. Our modified needles exhibited efficient electrocatalysis and high selectivity toward O_2_, with excellent repeatability. We well engineered a small diagnostic tool to monitor PO_2_, minimally invasive, able to monitor real-time O_2_ in vivo complex environments.

## 1. Introduction

Oxygen plays a critical role in physiological and pathological processes. Oxygen concentration is particularly important for viability in certain tissues such as the brain. A variety of brain disorders such as neural injury, brain dysfunction, vascular malformation, and neurodegenerative diseases are associated with abnormal levels of oxygen, whether it is hypoxia or even hyperoxia [1,2,3]. Furthermore, it has been well established that neurons in the brain are strongly affected by low levels of oxygen causing detrimental outcomes within minutes [4]. Current methods to directly monitor tissue oxygenation in the brain are varied. Non-invasive methods include functional Magnetic Resonance Imaging (fMRI), Positron Emission Tomography (PET), and near-infrared spectroscopy. These are expensive imaging techniques that allow for an overview, but have poor resolution, and suffer from a lack of accuracy as well as require transfer and even anesthesia of the patient [1,2,5]. Other direct techniques are invasive with the insertion of electrodes or probes. Electrodes are smaller than O_2_ detection probes. They are often based on the electrochemical detection of O_2_ on platinum (Pt) or carbon paste electrodes. Despite its invasiveness, this sensitive and cost-effectiveness method quickly provides a higher spatial resolution of a specific field of interest. To directly measure the partial pressure of oxygen (PO_2_) in brain tissue, large-sized electrodes such as the Clark electrode (Licox PbtO2 monitoring system, Integra Life Sciences Corporation, Plainsboro, New Jersey) are used [2,3]. The tip of that probe is fairly large (0.8 mm), which may cause detrimental brain damage [6]. Micro-size diameter electrodes with a high specific surface area and good sensitivity have been explored in diagnostics, for example, to monitor O_2_ levels in the body [7], but also for applications in drug delivery [8,9]. Beyond their accuracy, it is a promising alternative to measure and monitor oxygen in soft tissues while minimizing any additional pain, discomfort, and injury to the patient. One way to decrease the size of the electrode without affecting the electrochemical surface area, and therefore the sensitivity, is to use nanomaterials combined with a highly selective catalyst to prevent the need of incorporating an oxygen permeable membrane [10]. As an alternative catalyst to non-selective Pt, laccase, which is an oxidoreductase able to reduce oxygen into water, has been used in many process including developments of biosensor and biofuel cells [11,12]. These enzymes are highly specific to oxygen and the reduction reaction produces electrons that can be continuously monitored. Taking advantage of the selectivity of laccase for oxygen, this project focused on decreasing the interference to the electrode surface without the need for a selective membrane as a key to perform O_2_ monitoring. Minimizing the sensor to a needle-size diameter is an engineering method to obtain a sensor suitable for deep brain measurement [13,14]. In previous works, acupuncture needles (AN) have been modified to conduct electrical stimulus into deep tissue to evoke the contraction of musculature [15]. Acupuncture needles have also been modified with Fe(III)-porphyrin conjugated to functionalized graphene composites and used to monitor nitric acid release down to at the point of penetration in murine models [16]. Furthermore, functionalized needles coated with gold (Au) nanoparticles and a superficial graphene-oxide layer have been reported to successfully monitor dopamine in vitro and in vivo within the cerebral cortex of rats [17]. Acupuncture needles typically consist of medical-grade stainless steel alloys and have a minimal profile with the penetrative ability to reach deep tissues. While stainless steel is ineffective as a conductor, acupuncture needles may be etched and modified to act as electrochemical probes. Several strategies have been developed to create highly porous acupuncture needles. By varying the electrolyte, duration and potential surface of the needles can be altered following Fe removal [18,19,20,21] without undesired side effects or biocompatibility issues, however, some concerns about reproducibility have been raised. We propose acupuncture needles as a viable substrate for surface modification, and subsequent electrochemical study of oxygen monitoring. To develop a minimally invasive needle-sensor with a high surface area to monitor O_2_ levels in the brain, the approach was to directly etch the iron (Fe) from AISI 316L stainless steel acupuncture needles via a controlled pitting corrosion process, subsequently forming a high equivalent surface area (ESA) microporous surface morphology while only marginally altering their initial conductivity and probe diameter. In order to increase the conductivity and selectivity, we designed a robust three step approach for coating the pitted acupuncture needle substrate. This involves (1) the deposition of carbon nanoparticles using nonmagnetic stirring and nontoxic chemical; (2) adsorption of laccase; and (3) entrapment in an electrodeposited polypyrrole. The physicochemical properties of the materials have been thoroughly characterized as well as their efficacy and viability as probes for the electrochemical detection of PO_2_. 

## 2. Materials and Methods

Acupuncture needles (Seiren J-type, stainless steel AISI 316L 60 × 0.3 mm) were acquired from Eastern Currents Inc., Vancouver, BC, Canada. The DC power supply used to corrode the needles was manufactured by YaeCCC, (MS-305D). Ballistic gel (10% ballistic gelatin) was acquired from Clear Ballistics, Peterborough, ON, Canada. Sodium nitrate (NaNO_3_), potassium chloride (KCl), potassium ferricyanide (K_3_FeCN_3_), phosphate buffered saline (PBS) tablets, dimethyl sulfoxide (DMSO), ethanol, acetone, and ethylene glycol were purchased from Fisher Scientific, Ottawa, ON, Canada. Carbon nano-powder (mesoporous, >99.95%, <500 nm particle size), potassium nitrate (KNO_3_), ascorbic acid (C_6_H_8_O_6_), sodium sulfate (Na_2_SO_4_), laccase (*trametes versicolor*), sodium perchlorate (NaClO_4_), and pyrrole were purchased from Millipore Sigma, Oakville, ON, Canada. 

### 2.1. Preparation of Porous Acupuncture Needles (AN)

Stainless steel acupuncture needles (hereby referred to as ‘working electrodes’ or WE) were sequentially sonicated for 5 min at room temperature with acetone, 70% ethanol, and deionized H_2_O. Galvanic pitting corrosion was conducted using an electrolytic solution of 176 g/L NaNO_3_ in deionized H_2_O [17]. Corrosion trials were carried out in a two-electrode setup, with the AN-based electrodes serving as the cathode in conjunction with a glassy carbon (GC) anode. A thorough screening process was carried out, applying an array of potentials (ranging from 100 mV to 30 V) at various durations. Optimized properties include a maximized AN surface area via high micro pitted surface coverage, while only modestly thinning the AN diameter. Optimized trial conditions were uncovered with a direct potential of 5 V for a duration of 5 min with a sustained current range of 111–166 mA. Energy dispersive x-ray spectroscopy (EDX) was applied to confirm the mechanisms of pitting corrosion via a cross-comparison of the elemental constituent in pitted and unmodified probes. 

The carbon nanoparticle (CNP) coating of the unmodified acupuncture needle (UAN) and pitted acupuncture needle (PAN) electrodes was achieved using a novel spin dipping technique, as adapted from Merle et al., 2014 [22]. The CNP solution consisted of only 33.3 g/L carbon nanopowder, 30 mM ascorbic acid, and 0.1 M sodium sulfate in deionized water. The CNP solution was stirred at 1500 rpm for 4 h prior to dip coating. Each electrode was dipped in CNP solution, and then dried at ~65 °C for 15 min in an incubator. The electrode dipping step was repeated five times until a uniform carbon coating had formed. The resulting CNP coated needles were soaked overnight in a 1 mg ml-1 solution of laccase at 4 °C. After 24 h, a polypyrrole film was applied as a fastening layer to secure the CNP to the electrode. An electrolytic solution containing 0.1 M sodium perchlorate and 0.2 M of pyrrole monomer in PBS was prepared. Pyrrole monomer was electropolymerized via chronoamperometry at a potential of 0.855 V in a 3-electrode cell composed of the needle, platinum wire, and saturated calomel electrode (SCE) as the working, counter, and reference electrodes, respectively. Electrodeposition was stopped when the electric charge reached 250 mC.

### 2.2. Characterization of Porous Acupuncture Needles

Surface modifications were first optically verified under standard microscopy prior to characterization via scanning electron microscope (SEM), atomic force microscopy (AFM), Micro-computed tomography (Micro CT), and electrochemical analysis. Surface topography was analyzed using atomic force microscopy (DNP–Bruker, CM-AFM) using a conventional ACTA probe. Morphology study was conducted via micro CT (Skyscan 1172, version F) and EDX/SEM (FEI Inspect F50). The software CTVIX was used to generate micro CT images. The software ARgyle Light was used to generate 3D surface plots from raw AFM data files. Mechanical strength testing of all electrodes was achieved using an Instron 5567, with a traditional 100 N plate. Compressive strength testing of the UAN and PAN electrode subtypes was conducted using an Instron 5567 device. Ballistic gel (10% gelatin) was utilized to simulate human tissue in compressive strength testing, in order to gauge the penetrative ability of both common needle subtypes. Electrode tips (10 mm ± 1) were extracted and braced to the compressive plate using a conventional bracket. Compression test parameters were set to a rate of 0.1 mm/s using the 100 N compression plate.

Electrochemical analysis of modified acupuncture needle was achieved via cyclic voltammetry and chronoamperometry in a 0.1 M PBS buffer at pH 7.4 with a three-electrode setup, using a Pt-wire counter electrode (CE) versus a saturated calomel reference electrode (SCE RE). For electrochemical characterization, the CV setting entailed 10 cycles across a potential range of [−0.4/0.6 V] with a 5 mV/s scan rate in PBS pH 7.4 under nitrogen and air. O_2_ reduction performance and selectivity measurement were recorded in PBS via chronoamperometry at 0 V versus Ag/AgCl.

## 3. Results and Discussion

**Figure 1:**
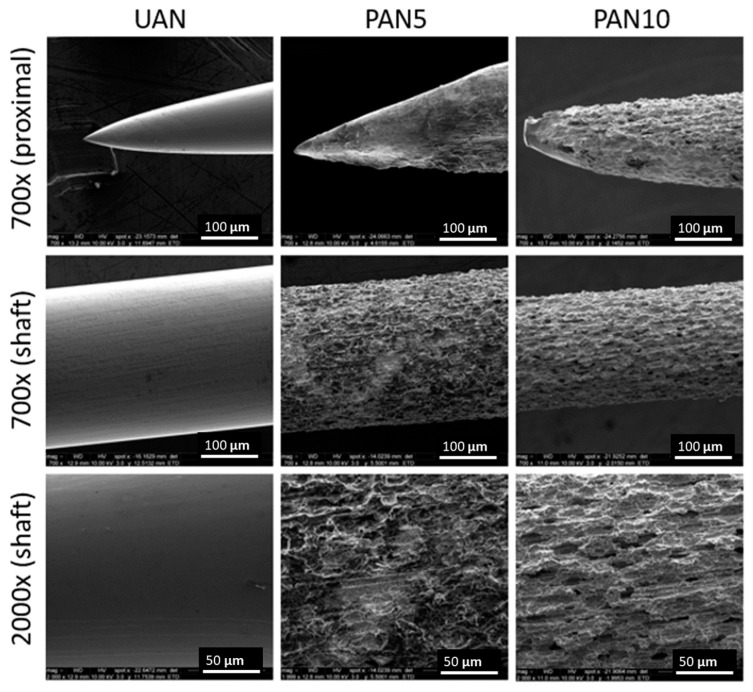
Scanning electron microscope (SEM) pictures of electrochemically pitted needles at 0, 5 and 10 min (UAN, PAN5, PAN10) at 700× (proximal and distal), and 2000× magnification via SEM.

The corrosion process was performed at various durations with the same potential. A corrosion treatment with a duration of more than 10 min led to a pronounced crack over the surface followed by a break. Figure 1 shows the SEM pictures of the surface of the stainless-steel acupuncture needles (UANs) and the pitted acupuncture needles (PANs). Corrosion trials were achieved using NaNO_3_ electrolyte in DI water at room temperature at 5 V anodization voltage and for different durations.

From Figure 1, it is apparent that the surface of UAN is smooth with a sharp tip whereas both PANs exhibit a rougher surface with various pores ranging from micro- to nanoscale. PAN10 exhibited a more uniform porosity along the shaft compared to the PAN5. The corrosion process created micro-pits with a distinct and deep open network. Additionally, the diameter of both PAN5 and PAN10 subtypes were reduced in contrast to the UAN probes, which were later quantified using micro CT to examine the extent of material loss.

Pitting corrosion is simply a localized form of corrosion, typically of an alloy of aluminum or stainless steel as a result of chemical or mechanical damage to the passivation film [23,24,25]. In our study, the UAN acted as the anode, the site at which metal oxidation occurs, resulting in the release of metal ions and free electrons. The free electrons reduce oxygen, thus providing a complimentary cathodic reaction [26]. Generally, the dissolution of the metal at the anode results in either the release of metal ions into the electrolytic solution, becoming hydrated, or the formation of a solid compound that collects on the surface as a result of metal ion release [27,28]. In the former case, further oxidation of the metal ions can occur and an open pit can form [28]. In the latter case, a protective barrier may be formed, and the collection of solid metal ions will inhibit further corrosion. Critical factors relating to the formation of pitting corrosion include the ionic strength and pH of the system as well as the complexing agents at play [29]. Furthermore, the composition of the particular alloy undergoing localized corrosion may alter the extent of metal release [30]. However, our study postulated that controlled pitting corrosion may be utilized as an effective electrochemical machining (ECM) method to produce highly porous surface topographies for biomedical application. The analysis of pitting corrosion in a quantitative manner poses a significant challenge as the heterogenous pits may form undercuts, distinct interconnected canal-like networks beneath the surface of a metal. As such, we rigorously characterized the pits via micro CT, AFM, and EDX/SEM to generate a holistic quantitative study of our ECM effect. Furthermore, the formation of pits consumes a small portion of the materials’ mass due to metal release and dissolution of surface oxide, which can result in mechanical degradation of the metal. With regard to this effect, it is imperative to gauge the mechanical integrity of the PAN-subtype probes to ensure their viability. EDX was used to identify changes between UAN and PAN subtypes to validate our theories of mechanism pertaining to the pitting corrosion of the SS probes (Supplementary Materials Figure S1). Fe composition for UAN probes were observed to mark 58.52 ± 0.82 w%, which reduced to 48.66 ± 1.37 w% among the PAN5 subtypes post-corrosion. Furthermore, upon pitting, we observed a mean loss of 9.86 w% Fe, thus supporting the theory of anodic Fe-release into the electrolytic solution during our controlled corrosion trials. Interestingly, a marked accumulation of +10.83% O in the PAN EDX scan supports the formation of an oxide film post-corrosion, indicating that a passivation layer formed as a secondary reaction initiated by our ECM approach. ImageJ (FIJI) software was used to better quantify/qualify the microporous structures. Analysis of SEM images via ImageJ (FIJI) provided approximations of pit size and distribution (Supplementary Materials Figure S2).

Quantification of surface porosity was accomplished using the open source image processing software ImageJ. Upon skeletonizing and augmenting image contrast to isolate porous formations, it can be approximated that 75% of the total surface across both pitting conditions with deeper pits covered 16% of PAN5s and 20% of PAN10s, respectively. Further quantification of 40 heterogenous micro-scaled pits ranging from ≥0.1 µm in diameter formed upon corrosion, in addition to ridges and depressions across the AN surface. Three separate regions (defined by a 300 × 300 px area) were drawn from both the PAN5 and PAN10 (2000×) SEM images, respectively, and were processed using ImageJ to isolate microstructures. Through this method, it has been assessed that PAN5 topography consists of an average pit diameter of 0.166 µm ± 0.09 whereas PAN10s consist of 0.25 µm ± 0.21 diameters, respectively. This marked alteration of surface topography suggests an augmented surface area in both pitted needle subgroups, with comparable total surface coverage. However, as the corrosion trial duration was increased from 5 to 10 min, we saw an increase in pit diameter rather than a noted increase in the number of total pits.

**Figure 2:**
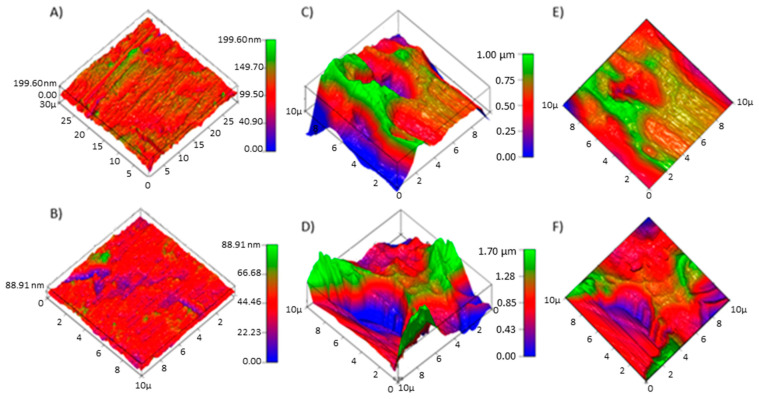
AFM-generated 3D surface profiles, scanning a 10 µm^2^ area of UANs (**A**,**B**), PAN5 (**C**,**E**), and PAN10s (**D**,**F**). Surface plotting provided a schematic illustration of surface roughness and was applied in conjunction with the AFM data as displayed in Table S1.

AFM was subsequently applied to better qualify the surface topography and provide analysis for roughness factors and localized microporous formation (Figure 2). Electrode profiles were analyzed via AFM through scanning a 10 µm^2^ area and through mapping contact at the AFM cantilever/sample interface.

All scans successfully covered the same scan size (10 µm^2^), mapping the same number of points (262,144) regardless of the sample type, and providing a complete profile as all samples achieved an XY% of 100. It was determined that while the scan size is quite small relative to the total surface, it allows for a comprehensive analysis of localized topography representative of the entire electrode sample. However, due to the innate roughness of pitted Ans, the cantilever was prone to sticking and snapping back on surface ridges skewing the max-z, min-z, and root mean squared (RMS) values. Despite this effect, we can infer that the effect of pitting corrosion drastically alters electrode morphology through increasing the depth of pits and roughness of surface artifacts. Nonetheless, surface area (SA) and RMS stand as reasonable markers for surface morphology, if only on a relative scale with reference to the UAN samples. It is critical to note that at this point of the experimental process, PAN10s were abandoned due to their complex characterization and issues relating to reproducibility despite their increased roughness and SA. PAN10s were prone to excess material loss causing mechanical deficits such as total corrosion and snapping of the electrode shaft.

**Figure 3:**
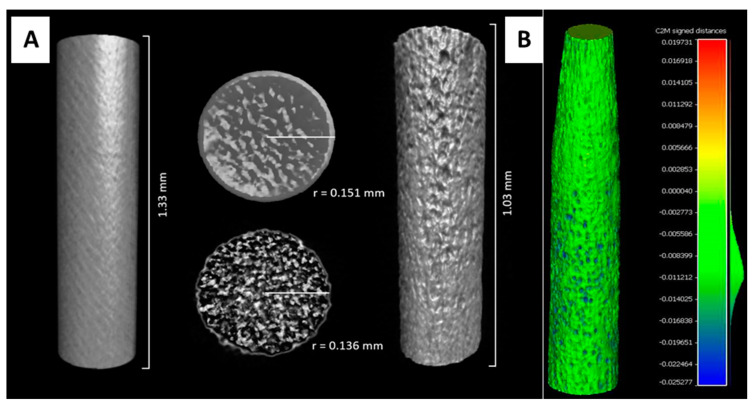
Micro CT images generated with CTVOX software (**A**) and Cloud Compare software (**B**). mage A depicts UAN vs. PAN5 change in diameter as a result of pitting corrosion, with material loss of 0.015 mm. Image B confirms the material loss across the entire PAN5 surface, following a normal distribution ranging from 0.08–0.011 mm.

Micro CT was further applied to determine material loss, particularly the thinning of AN diameters post-corrosion among the PAN subtypes. We observed a homogenous material loss across PAN5 in comparison to the UAN samples, which was shown to follow a normal distribution ranging from 0.008–0.011 mm (Figure 3). Furthermore, Cloud Compare results were validated with a simplistic CT measure showing a loss of 0.015 mm in PAN5 diameter at the midpoint.

Compressive testing of both UAN and PAN subtypes was conducted to qualify the mechanical integrity and penetrative ability of the needles into simulated tissue. UAN and PAN subtypes show distinct yet stereotyped averaged curves with standard deviation denoted by error bands (Supplementary Materials Figure S3). Neither group showed any bending or breaking when penetrating the ballistic gel, thus suggesting their viability for deep-tissue application. Furthermore, these tests confirmed that the material loss and subsequent thinning of the PAN subtype caused no compromise to their integrity versus the UANs. Slight snagging on the ballistic gel in PAN subtype was observed as a mere indication of the roughness of their surface morphology. Interestingly, despite the roughness of the PANs, they in fact required a lower corresponding load average per unit displacement in contrast. As such, the mechanical tests performed suggest that not only do the PANs maintain robust properties after pitting, they require lower force to penetrate the simulated tissue in comparison to the conventional UANs.

**Figure 4:**
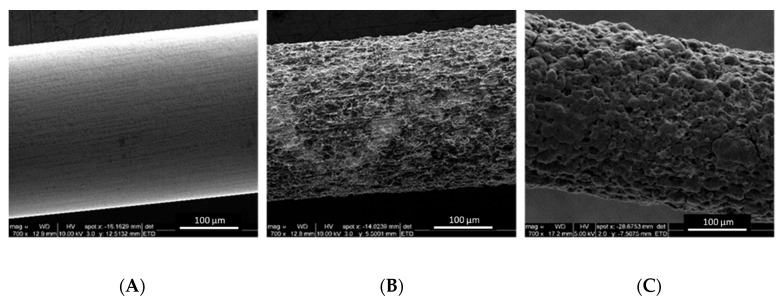
SEM images of (**A**) UAN, (**B**) PAN5, and (**C**) Lacc/CNP/PPy coated PAN at 700× magnification.

Prior to application, PAN electrodes were further modified with carbon nanoparticles, enzymes, and polypyrrole. The needles were dip coated with carbon nanoparticles (CNP) following a protocol developed by Merle et al. [22]. Ten dipping/drying cycles were required to homogenously cover the surface of the needle. The CNP/PAN electrodes were then soaked in an enzyme solution (Lacc) overnight. The next day, a polypyrrole (PPy) film as a conductive bioadhesive was electrodeposited on the surface of the Lacc/CNP/PAN electrodes to prevent nanomaterials and biocatalysts from detaching and leaching out in the body. Figure 4 shows the SEM images of the conventional needle (UAN), the pitted needle (PAN), and finally the lacc/CNP/PPy coated PAN. From Figure 4, it is obvious that UAN exhibits a smooth surface, while PAN possesses micro-and nanoscale pores. However, the lacc/CNP/PPy coated PAN had a distinct topology with agglomerates covering the porosity of the PAN, creating a rough surface and macroscale pores. To confirm that the polypyrrole strategy provides a strong physical adhesion of the carbon nanoparticles on the needles while providing a stable microenvironment for the enzyme, SEM analysis was carried out before and after insertion in the ballistic gel (Figure S4).

**Figure 5:**
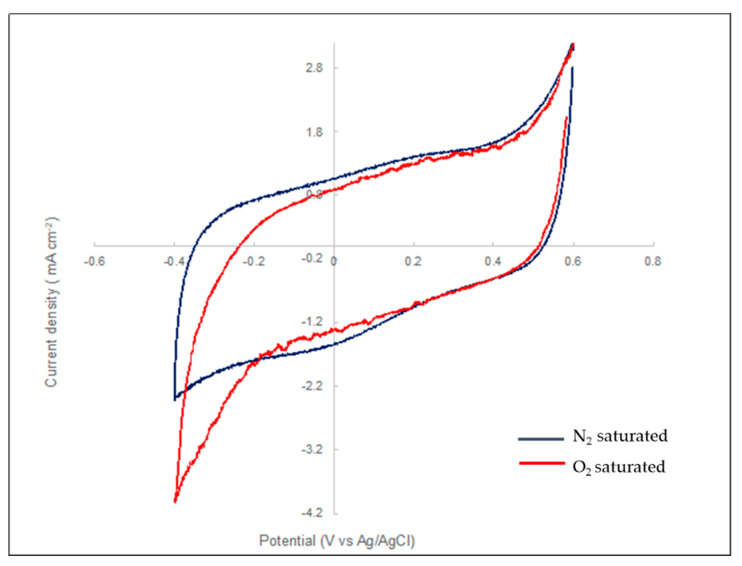
Cyclic voltammograms of Lacc-CNP-PPy/PAN electrode. The experiments were performed in 0.1 M phosphate buffer, pH 7.5, in nitrogen (blue) and air saturated (red) solutions at scan rates of 5 mV s^−1^.

To determine whether it could monitor effectively oxygen, electrochemical measurements were performed. For the lacc/CNP/PPy coated PAN electrode to record the oxygen reduction, laccase must be able to directly transfer (without a mediator) the electrons from the electrodes to the oxygen via the active site of the enzyme. To determine whether laccase attached to the electrode is able to catalyze the reaction and act as a molecular transducer, the first cyclic voltammetry was performed. Figure 5 is the current–potential curves at both electrodes in a nitrogen and oxygen-saturated PBS buffer at 37.5  °C.

Under anaerobic conditions, the laccase-modified PAN electrode exhibited cyclic voltammograms that were indistinguishable from the control voltammograms in the absence of the enzyme (Supplementary Materials Figure S5). CV shows a quasi-reversible electron transfer process with a midpoint potential of 105 mV versus Ag/AgCl typical from the T1 active site of the enzyme. It is shown that the T1 site of the laccase is the primary electron acceptor and that the electron can be directly transferred from solution to the surface of the electrode without the use of a mediator. 

**Figure 6:**
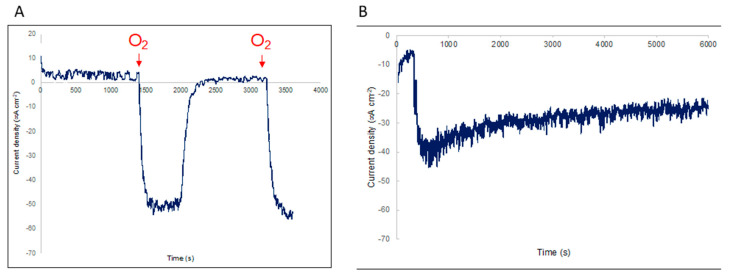
(**A**) Electrocatalytic oxygen reduction performance at the Lacc-CNP-PPy/PAN electrode in PBS solution (pH 7.4) at room temperature. Durability and selectivity measurement of the Lacc CNP-PPy/PAN electrode (**B**). Oxygen addition at 400 s followed by gamma-aminobutyric acid (GABA) and catechol at 2000 s and 4000 s, respectively.

The Lacc-CNP-PPy/PAN electrode was employed for the electrocatalytic reduction of O_2_. The laccase enzymes wired by the carbon nanoparticles to the PAN were used as bioelectrocatalysts. The electrochemical performances of the Lacc-CNP-PPy/PAN toward oxygen electroreduction were investigated by chronoamperometric measurements in PBS in N_2_ and O_2_ saturated solutions. As shown in Figure 6A, the lacc/CNP/PPy/PAN electrode displayed excellent electrochemical reduction behavior to O_2_ and showed a current density of 50 μA cm^−2^ while AN, PAN, and CNP/PPy/PAN showed no obvious current response.

This confirmed the key catalytic role of laccase toward O_2_. The measured current densities were recorded as −3.18 ± 1.31 and −49.12 ± 3.73 between Lacc-CNP-PPy/PAN and the counter electrodes for 2% and 96% of dissolved oxygen, respectively. Each data point in the plot is the average of chronoamperometry current after the sensor reached a stable value (Figure S6A). A linear fit was given by current density (μA cm^−2^) = −0.4887 * O_2_ concentration −2.2026 over the oxygen range, which demonstrates a sensitivity of −0.49 μA cm^−2^ per % PO_2_ of the sensor. It is worth nothing that not only did the lacc/CNP/PPy/PAN electrode show great reduction capability, but also excellent repeatability (Figure S6B). Moreover, we investigated the selectivity of the lacc/CNP/PPy/PAN electrode toward some common interfering molecules found in brain tissues such as gamma-aminobutyric acid (GABA) and catechol. As showed in Figure 6B, the current density decreased at 400 s due to the addition of O_2_ in the solution, but did not change upon the addition of GABA and catechol at 2000 s and 4000 s, respectively. From Figure 6B, it is obvious that the lacc/CNP/PPy/PAN electrode had a distinct response to O_2_. All the above results demonstrate that the as prepared lacc/CNP/PPy/PAN electrodes have remarkable electrocatalytic performance and excellent selectivity to O_2_ (Supplementary Materials Table S2). Given that PO_2_ in a healthy brain is ~30–48 mm Hg and could potentially reach 10 mmHg when traumatic brain injury is suspected, the lacc/CNP/PPy/PAN needle, having been tested in a dynamic range between 10 mmHg and 700 mmHg, could potentially be used to monitor brain tissue oxygenation clinically.

## 4. Conclusions

In conclusion, we designed and engineered a functional acupuncture needle based on laccase and carbon nanoparticles entrapped in a biocompatible conductive adhesive for real-time and in vivo monitoring of O_2_ with remarkable performances for potential brain tissue application. The lacc/CNP/PPy/PAN electrodes exhibited efficient electrocatalysis and high selectivity toward O_2_, allowing the real-time monitoring of O_2_ in complex environments. Future works include biofouling assessment and drift measurement, especially when long term recording is required, for example, a 7-day measurement is usually required for patients suffering from shunt malfunctions. Using a minimally invasive functional acupuncture needle as a simple electrode for in vivo electrochemical measurement is relevant because there is a need for a small diagnostic tool to monitor PO_2_ with accuracy and free from complications for a wide spectrum of injuries to the brain.

## Supplementary Materials

The following are available online at https://www.mdpi.com/2079-6374/10/11/157/s1, Figure S1: EDX analysis of UAN (left) and PAN5 (right), with four selected regions of analysis to contract weight% and shifts in elemental constituents prior to and post electrochemical pitting corrosion, Figure S2: Schematic of SEM-image treating using FIJI software to characterize porosity (a) SEM images of PAN5 and PAN10 (2000×), (b) snipped to a 300 × 300-pixel area and skeletonized, with 40 ‘pits’ identified and measured to scale. Resulting measure of porosity found PAN5 and PAN10 average pit size to be 0.166 µm ± 0.09 and 0.25 µm ± 0.21, respectively, Figure S3: Instron graph detailing the average load for both UAN and PAN subtypes (n = 3, respectively) while compressing into ballistic gel. Initial penetration of ballistic gel achieved at ~5mm of displacement from the 100 N force plate. Error bands mark the standard deviation of the moving load averages for both groups, Figure S4: SEM pictures (A) before and (B) after simulating the injection of the Lacc-CNP-PPy/PAN needle in ballistic gel, Figure S5: Cyclic voltammograms of the CNP-PPy/PAN electrode. The experiments were performed in 0.1 M phosphate buffer, pH 7.5, in a nitrogen solution at scan rates of 5 mV s-1, Figure S6: (A) Plot of the calibrated O_2_-concentrations function of the measured current across the Lacc-CNP-PPy/PAN electrode and counter electrode. (B) Electrocatalytic oxygen reduction performance at three different Lacc-CNP-PPy/PAN electrodes in PBS solution (pH 7.4) at room temperature, Table S1: Atomic force microscopy data, displaying measures of physical characterization such a range (max, min) along the z-axis, RMS, SA, scan size for 10 µm^2^ for the UAN, PAN5, and PAN10 electrode sample, Table S2: Comparative study showing the efficiency of the Lacc-CNP-PPy/PAN electrode.
