# Supplementary material for: Microelectrochemical Smart Needle for Real Time Minimally Invasive Oximetry
Source: Biosensors (Basel). 2020 Oct 29;10(11):157. doi: 10.3390/bios10110157 (PMC7693384; doi:10.3390/bios10110157)
Supplement: Supplementary file 1 [file biosensors-10-00157-s001.pdf]

biosensors

Article

Microelectrochemical Smart Needle for Real Time Minimally Invasive Oximetry

Daniela Vieira [1], Francis McEachern [1], Romina Filippelli [1], Evan Dimentberg [1], Edward J Harvey [2] and Geraldine Merle [2,3]*

1 Experimental Surgery, Faculty of Medicine, McGill University, Montreal, QC H3A 0C5, Canada; daniela.vieira@mail.mcgill.ca (D.V.); francis.mceachern@mail.mcgill.ca (F.M.); romina.filippelli@mail.mcgill.ca (R.F.); Evan.dimentberg@mail.mcgill.ca (E.D.)
2 Department of Surgery, Faculty of Medicine, McGill University, Montreal, QC H3A 0C5, Canada; edward.harvey@mcgill.ca
3 Chemical Engineering Department, Ecole Polytechnique de Montréal, P.O. Box 6079 Station, Montreal, QC H3C 3A7, Canada
* Correspondence: Geraldine.merle@polymtl.ca; Tel.: +1-514-340-4711 (ext. 3667)

Received: 15 September 2020; Accepted: 27 October 2020; Published: date

Supplementary Materials

.

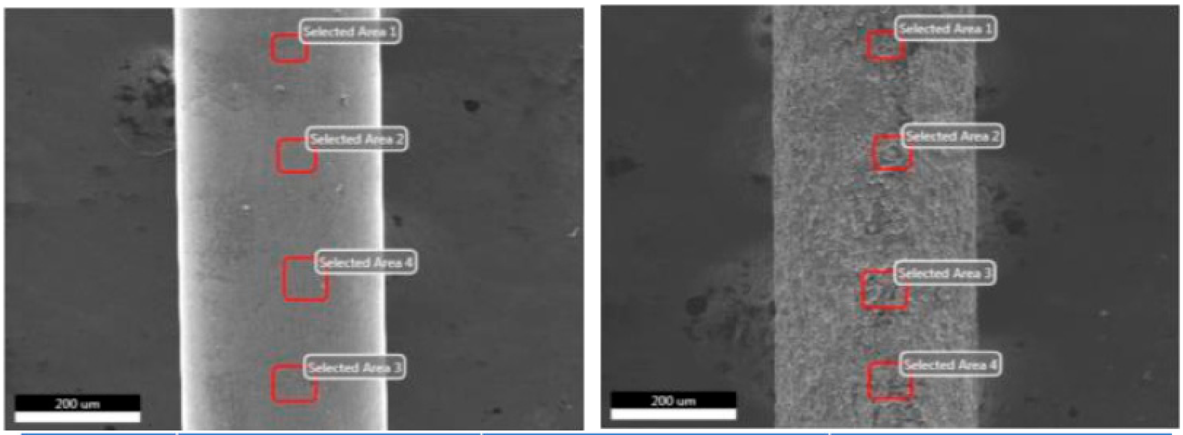

Element	UAN Weight% (SD)	PAN5 Weight % (SD)	ΔWeight%
Fe	58.52 (±0.80)	48.66 (±1.37)	-9.86
Cr	28.54 (±0.1.34)	27.81 (±2.39)	-0.7225
C	9.23 (±0.69)	5.85 (±0.96)	-3.37
O	0.25 (±0.23)	11.08 (±1.71)	10.83
Si	1.54 (±0.13)	0.64 (±0.03)	-0.9

Figure S1. EDX analysis of UAN (**left**) and PAN5 (**right**), with four selected regions of analysis to contract weight% and shifts in elemental constituents prior to and post electrochemical pitting corrosion.

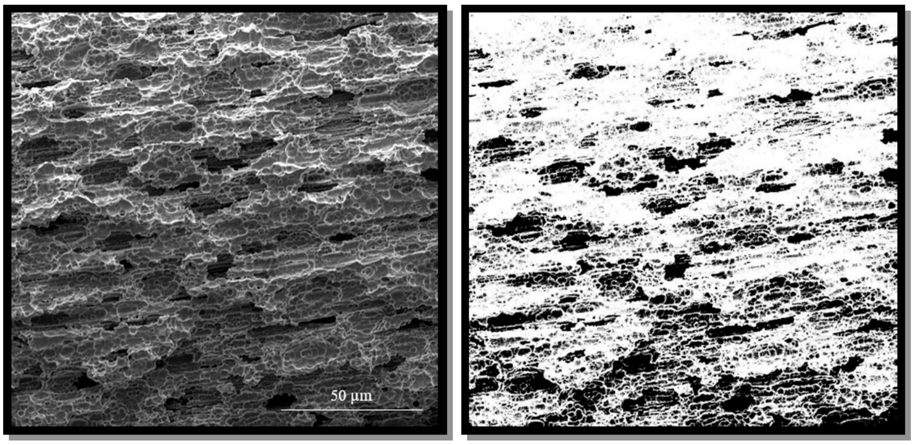

Figure S2. Schematic of SEM-image treating using FIJI software to characterize porosity. (**a**) SEM images of PAN5 and PAN10 (2000×), (**b**) snipped to a 300 × 300-pixel area and skeletonized, with 40 'pits' identified and measured to scale. Resulting measure of porosity found PAN5 and PAN10 average pit size to be 0.166 µm ± 0.09 and 0.25 µm ± 0.21, respectively.

Table S1. Atomic force microscopy data, displaying measures of physical characterization such a range (max, min) along the z-axis, RMS, SA, scan size for 10 µm^2 for the UAN, PAN5, and PAN10 electrode sample.

AFM DATA	UAN	UAN (2)	PAN5	PAN10
**NUMBER OF POINTS**	262,144	262,144	262,144	262,144
**MAX-Z (µM)**	32.65	48.03	738.02	1682.00
**MIN-Z (µM)**	22.92	38.29	908.032	1127.00
**RMS (µM)**	5.45	7.54	224.37	270.343
**PERCENT XY (%)**	100	100	100	100
**SA (µM)**	100.1	100.4	117.9	159.6
**SCAN SIZE (µM2)**	10	10	10	10

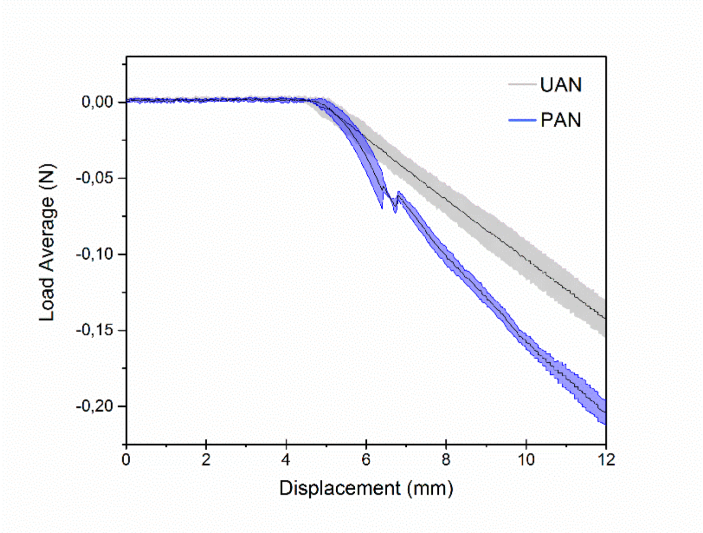

Figure S3. Instron graph detailing the average load for both UAN and PAN subtypes (n = 3, respectively) while compressing into ballistic gel. Initial penetration of ballistic gel achieved at ~5mm of displacement from the 100 N force plate. Error bands mark the standard deviation of the moving load averages for both groups.

Table S2. Comparative study showing the efficiency of the Lacc-CNP-PPy/PAN electrode.

Material	Current density (O2 saturated) (mA/cm2)	Electrode size (mm)
Lacc-CNP-PPy/PAN (this work)	−4.2	0.3
Carbon microfiber - Co/N/C [1]	−1	0.15
AuragenTM Depth Electrode (Pt) [2]	−0.35	1.2
Pt/Nafion [3]	−2.16	0.5

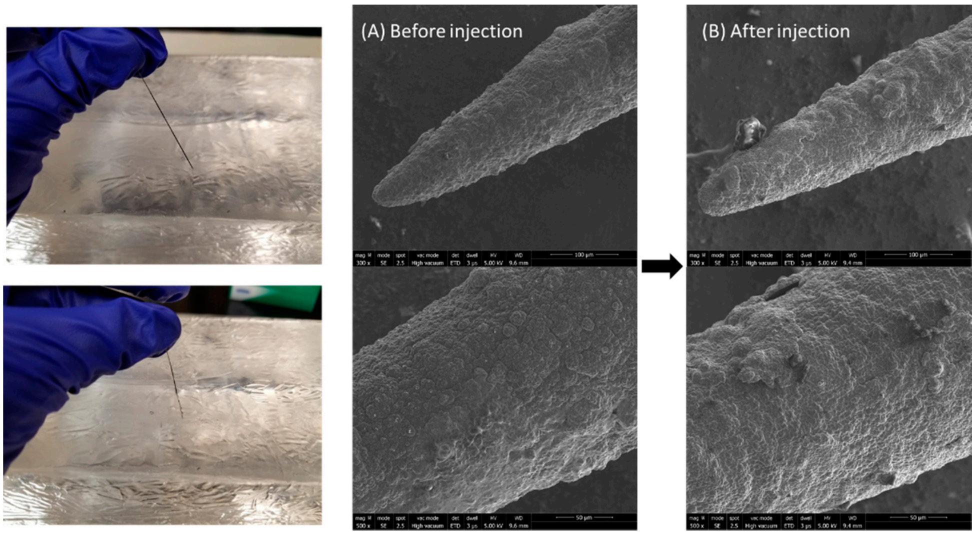

Figure S4. SEM pictures (**A**) before and (**B**) after simulating the injection of the Lacc-CNP-PPy/PAN needle in ballistic gel.

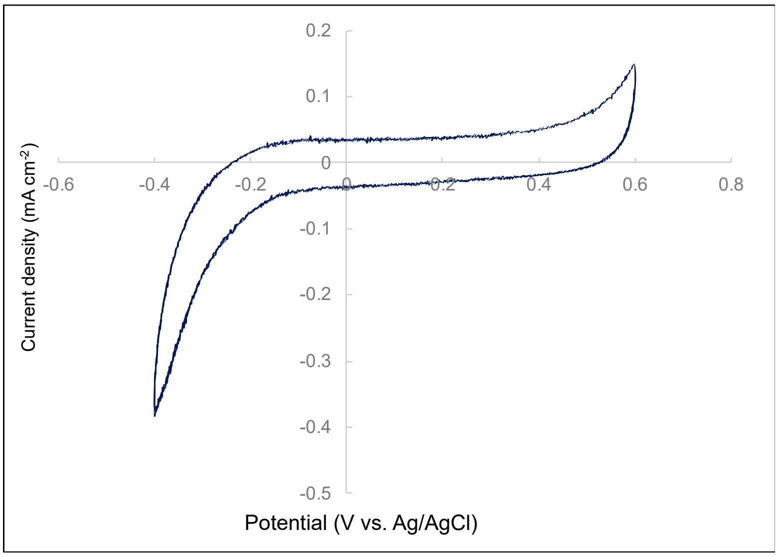

Figure S5. Cyclic voltammograms of the CNP-PPy/PAN electrode. The experiments were performed in 0.1 M phosphate buffer, pH 7.5, in a nitrogen solution at scan rates of 5 mV s-1.

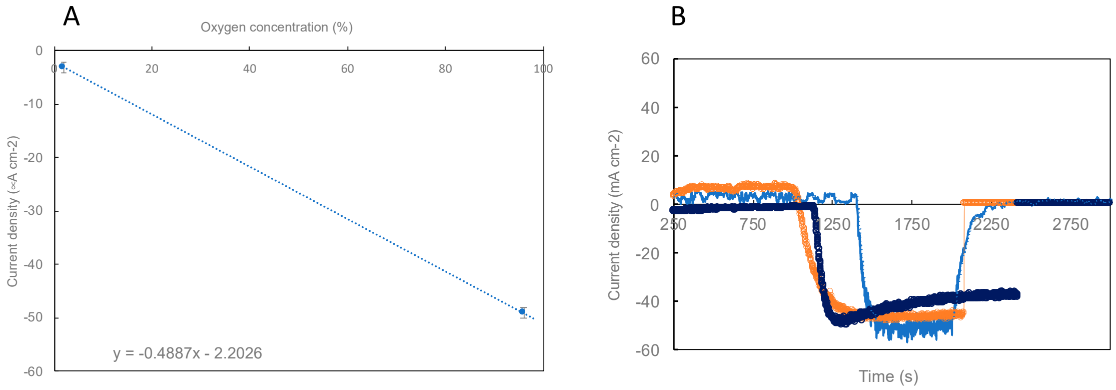

Figure S6. (**A**) Plot of the calibrated O_2-concentrations function of the measured current across the Lacc-CNP-PPy/PAN electrode and counter electrode. (**B**) Electrocatalytic oxygen reduction performance at three different Lacc-CNP-PPy/PAN electrodes in PBS solution (pH 7.4) at room temperature.

References

1. Cao, Y.; Ma, W.; Ji, P.; Yu, F.; Wu, H.; Wu, L. Electrophoretically Sheathed Carbon Fiber Microelectrodes with Metal/Nitrogen/Carbon Electrocatalyst for Electrochemical Monitoring of Oxygen in Vivo. *ACS Appl. Biol. Mater.* **2019**, *2*, 1376.
2. Ledo, A.; Fernandes, E.; Quintero, J.E.; Gerhardt, G.A.; Barbosa, R.M. Electrochemical evaluation of a multi-site clinical depth recording electrode for monitoring cerebral tissue oxygen. *Micromachines* **2020**, *11*, 632.
3. Rivas, L.; Dulay, S.; Miserere, S.; Pla, L.; Marin, S.B.; Parra, J.; Eixarch, E.; Gratacós, E.; Illa, M.; Mir, M. Micro-needle implantable electrochemical oxygen sensor: ex-vivo and in-vivo studies. *Biosens. Bioelectron.* **2020**, *153*, 112028.
